# Natural Phenolics Disrupt Microbial Communication by Inhibiting Quorum Sensing

**DOI:** 10.3390/microorganisms13020287

**Published:** 2025-01-27

**Authors:** Martin Helcman, Karel Šmejkal, Marie Čulenová, Tibor Béres, Jakub Treml

**Affiliations:** 1 Department of Natural Drugs, Faculty of Pharmacy, Masaryk University, 612 00 Brno, Czech Republic; helcmanm@pharm.muni.cz (M.H.); smejkalk@pharm.muni.cz (K.Š.); culenovam@pharm.muni.cz (M.Č.); 2 Faculty of Sciences, Palacký University, 779 00 Olomouc, Czech Republic; tibor.beres@upol.cz; 3 Department of Molecular Pharmacy, Faculty of Pharmacy, Masaryk University, 612 00 Brno, Czech Republic

**Keywords:** antibacterial, *Cannabis sativa*, *Morus alba*, phenolic, prenyl, quorum sensing

## Abstract

Quorum sensing, a bacterial cell-to-cell communication mechanism, plays a key role in bacterial virulence and biofilm formation. Targeting quorum-sensing pathways represents a promising strategy for the development of novel antibacterial agents. This study evaluated the anti-quorum-sensing activities of 18 natural compounds, including cannabinoids, arylbenzofurans, flavonoids, caffeine, and chlorogenic acid, using the luminescent biosensor strain *Vibrio harveyi* MM30. *V. harveyi* MM30, a mutant strain deficient in the production of autoinducer-2 (AI-2) but responsive to exogenous AI-2, was used to assess the activity of test compounds on the AI-2 receptor pathway. Test compounds were incubated in AI-2-containing media, and luminescence was measured to evaluate quorum-sensing inhibition. Comparisons were made in the absence of AI-2 to determine AI-2-independent inhibitory activity. The most active compounds were further tested on methicillin-resistant *Staphylococcus aureus* (MRSA 7112) to determine their effects on AI-2 production in spent media. Among the tested compounds, the non-prenylated arylbenzofuran moracin M and the prenylated arylbenzofuran moracin C exhibited significant quorum-sensing inhibitory activity in the AI-2-mediated pathway. None of the test compounds significantly inhibited quorum sensing in the absence of AI-2. Five compounds (cannabigerol, cannabidiol, cannabigerolic acid, moracin M, and moracin C) were selected for further investigation in MRSA 7112 cultures. The spent media from MRSA 7112 cultures treated with moracin M (16, 32, 64 µg/mL) and cannabigerolic acid (16 µg/mL) showed significant inhibition of AI-2 production when transferred to *V. harveyi* MM30 cultures. Moracin M and cannabigerolic acid demonstrated potential as quorum-sensing inhibitors by targeting AI-2 production and signalling pathways in MRSA 7112 and *V. harveyi*. These findings suggest their potential for further development as antibacterial agents targeting quorum-sensing mechanisms.

## 1. Introduction

Quorum sensing (QS) serves as a cellular communication mechanism employed by microbes to control, e.g., virulence and biofilm formation. During growth, cells release autoinducers, small and diffusible compounds that accumulate in the environment [1]. Their release rate is increased by signal molecules, resulting in positive feedback to cells present at elevated densities and a substantial increase in cooperative efforts. QS offers a mechanism for individual bacteria to gauge local cell density and engage in cooperation once a threshold density is achieved [2]. The concept of QS was first introduced by Fuqua et al. to describe the population-density-dependent regulation of bioluminescence in the Gram-negative marine bacterium *Aliivibrio fischeri* [3]. The related species *Vibrio harveyi*, a common pathogen of marine animals, is often used as a QS biosensor strain because its light production can be easily quantified [4].

The bioluminescence in this bacterium is regulated through the production, build-up, and self-recognition of specific signalling molecules, including autoinducer 1 (AI-1), autoinducer 2 (AI-2), and *Vibrio cholerae* autoinducer 1 (CAI-1) [5]. The signalling molecule of the AI-1 system in *V. harveyi* has been characterized as hydroxybutanoyl-L-homoserine lactone [6]. The AI-2 in *V. harveyi* was discovered to be furanosyl borate diester [5], and its precursor was identified as (*S*)-4,5-dihydroxypentane-2,3-dione [7]. CAI-1 was identified as (*S*)-3-hydroxytridecan-4-one [8]. Nitric oxide (NO) also plays a role in light production, flagellar production, and the promotion of biofilm formation. NO is therefore sometimes considered to be the fourth quorum-sensing autoinducer molecule of this bacterium [9].

Whereas the production of AI-1 is considered to be species-specific, the production of AI-2 has been demonstrated in numerous bacterial species of both Gram-negative and Gram-positive bacteria [10]. This observation has given rise to the hypothesis that AI-2 serves as a means of interspecies communication [2].

In *V. harveyi*, either the AI-1 system or the AI-2 system alone can control the density-dependent expression of luminescence [11]. Our objective was to evaluate whether the compounds under study have the ability to interact with these systems and potentially influence their luminescence output. Several genetic mutant strains of *V. harveyi* are known and utilized for specialized bioluminescence assays.

In contrast to traditional antibiotics, quorum-sensing inhibitors (QSIs) functioning as anti-virulence agents aim to diminish virulence without inhibiting bacterial growth. Consequently, anti-QS therapies could mitigate the evolutionary pressure on bacterial populations to develop resistance [12]. The strategy of blocking QS may disarm pathogens, making them highly susceptible to elimination by the immune system or lower doses of antibiotics [13]. QSI activity has been described in many classes of plant products, including flavonoids [14], furanocoumarins [15], terpenoids [16], alkaloids [17], and phenylpropanoids [18]. Flavonoids are known to inhibit QS by blocking the autoinducer-binding receptors, LasR and RhlR. Structure–activity relationship studies reveal that two hydroxyl groups in the flavone A-ring backbone are essential for effectively inhibiting these receptors. Biochemically, flavonoids act non-competitively, preventing LasR/RhlR from binding to DNA. When applied to *P. aeruginosa*, flavonoids alter the transcription of genes controlled by QS and reduce the production of virulence factors [19]. Anti-quorum-sensing activity has been previously reported, not only for the naturally occurring phytocannabinoid cannabigerol [12], but also for synthetic analogues of cannabinoids [20] and endocannabinoids [21].

**Figure 1:**
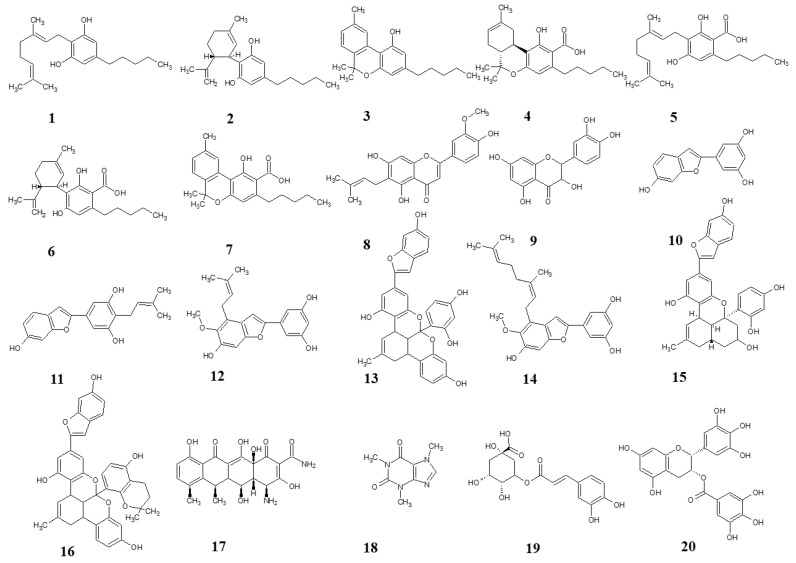
Structures of test compounds. Cannabigerol (**1**), cannabidiol (**2**), cannabinol (**3**), tetrahydrocannabinolic acid (**4**), cannabigerolic acid (**5**), cannabidiolic acid (**6**), cannabinolic acid (**7**), cannflavin B (**8**), quercetin (**9**), moracin M (**10**), moracin C (**11**), moracin T (**12**), albanol B (**13**), mulberrofuran Y (**14**), mulberrofuran G (**15**), mulberrofuran K (**16**), doxycycline (**17**), caffeine (**18**), chlorogenic acid (**19**), epigallocatechine gallate (**20**).

We evaluated the QS inhibition properties of various natural prenylated and non-prenylated phenolics, such as cannabinoids, arylbenzofurans, and flavonoids (Figure 1). In contrast to cannabinoids and flavonoids, arylbenzofurans have not been documented to show anti-QS activity, to this day. Nonetheless, because the furan structure is found in various recognized natural quorum-sensing inhibitors [22], we decided to explore the potential of these molecules [23]. Furthermore, in our search for a positive control for the bioluminescence assay, we tested three compounds previously reported in the literature for their specific anti-QS activity: caffeine [24], epigallocatechin gallate [25], and chlorogenic acid [26]. The previously demonstrated anti-QS activity of these compounds—although not in this specific assay or in these bacterial species—made them relevant candidates for our study. Initially we assessed the antibacterial activity of our compounds against *Vibrio harveyi* MM30 and methicillin-resistant *Staphylococcus aureus* (MRSA) 7112. Subsequently, subinhibitory concentrations of these compounds were employed in three distinct bioluminescence assays to evaluate their effects on QS mechanisms. Specifically, we examined the inhibition of AI-2 regulated QS and AI-2 independent QS in *V. harveyi* MM30, as well as AI-2 production in MRSA 7112.

## 2. Materials and Methods

### 2.1. Plant Material

A tetrahydrocannabinol-rich variety of *Cannabis sativa* L. (Cannabaceae) was kindly donated by the Czech University of Life Sciences in Prague. The cannabigerol- and cannabidiol-rich cultivars of *C. sativa* were both generously supplied by the Cannilav (Brno, Czech Republic).

### 2.2. Chemicals

#### 2.2.1. Isolation of Plant Chemicals

The above-mentioned drugs were subjected to a series of purification steps, including ethanol extraction, liquid–liquid extraction, column chromatography, flash chromatography with the use of flash column chromatography (PuriFlash 5.205 apparatus (Interchim, Montluçon, France) and semipreparative high-performance liquid chromatography (Dionex UltiMate™ 3000 with UV/vis detection; ThermoFisher Scientific, Waltham, MA, USA). For the identification of isolated compounds, we used analytical HPLC Agilent 1100 Series with UV/Vis detection (Agilent Technologies, Santa Clara, CA, USA) and mass spectrometry detector (TSQ Quantum Access Max triple quadrupole (ThermoFisher Scientific)). For details, please see supporting info (Figures S1–S10, Tables S1–S3)

Cannabigerol (**1**), cannabinol (**3**), tetrahydrocannabinolic acid (**4**), cannabinolic acid (**7**), and cannflavin B (**8**) were isolated from a tetrahydrocannabinol-rich variety of *Cannabis sativa* L. (Cannabaceae). Cannabigerolic acid (**5**) and cannabidiolic acid (**6**) were isolated from cannabigerol- and cannabidiol-rich varieties of *Cannabis sativa* (respectively).

Moracin M (**10**), moracin C (**11**), albanol B (**13**), mullberofuran Y (**14**), and mullberofuran G (**15**) were obtained from *Morus alba* L. (Moraceae) root bark, as described previously [27].

#### 2.2.2. Purchased Chemicals

Chemicals were purchased as follows: cannabidiol (**2**) from Knowde (USA), quercetin (**9**) from Koch-Light laboratories Ltd. (Suffolk, UK), moracin T (**12**) and mullberofuran K (**16**) from ChemFaces (Wuhan, China), doxycycline (**17**) from Fagron (Olomouc, Czech Republic), caffeine (**18**) from Lancaster (Morecambe, UK), chlorogenic acid (**19**), and epigallocatechin gallate (**20**) from Sigma-Aldrich (St. Louis, MO, USA).

Mueller–Hinton medium and microbial agar were obtained from Sigma-Aldrich. QS AI-2 Bioassay (AB) medium was composed of NaCl (Penta, Praha, Czech Republic), MgSO_4_·7H_2_O (Lach-Ner, Neratovice, Czech Republic), casamino acids (Gibco-Life technologies corporation—Thermo Fisher, Waltham, MA, USA), KOH (Sigma-Aldrich, St. Louis, MO, USA), L-arginine and thiamine (VWR Chemicals, Radnor, PA, USA), riboflavin (Sigma-Aldrich, St. Louis, MO, USA), KH_2_PO_4_ (Penta, Praha, Czech Republic), K_2_HPO_4_ (Penta, Praha, Czech Republic), and glycerol (Sigma-Aldrich, St. Louis, MO, USA).

Preparation of AB (AI-2 bioassay) medium: 300 mM NaCl, 0.5 mM MgSO₄, and 2mg/mL of casamino acids were mixed with distilled water. KOH was added to adjust the pH to 7.5. The mixture was then autoclaved for 30 min. A total of 0.5 mM L-arginine, 20 μg/mL thiamine, 2 μg/mL riboflavin, 2.5 mM K_2_HPO_4_, 2.5 mM KH_2_PO_4_ and 0.5% glycerol were each dissolved separately, in distilled water. These solutions were filtered through a 0.2 μm microfilter syringe and subsequently added to the rest of the AB medium for *V. harveyi* MM30 [14].

### 2.3. Bacterial Strains

The *Vibrio harveyi* mutant strain MM30 was kindly donated by the Department of Food Science and Technology, Biotechnical Faculty, University of Ljubljana [28]. The *V. harveyi* strain MM30 is deficient in the *luxS* gene, and therefore cannot synthesize its own AI-2 [11,29]. Although the AI-1 system remains functional and capable of independently generating luminescence [30], the intensity of the light produced is significantly lower than that observed with the addition of exogenous AI-2. This strain is widely used to evaluate AI-2 production in other bacteria [28], and is also useful for determining the activity of test compounds on the LuxPQ receptor, including the inhibition or induction of AI-2 binding to the receptor site [12].

The methicillin-resistant *Staphylococcus aureus* (MRSA) strain 7112 was purchased form the Czech Collection of Microorganisms, Masaryk University, Brno. *Staphylococcus aureus* bacteria, in general, are known to produce AI-2 molecules [31].

### 2.4. Determination of Minimal Inhibitory Concentration (MIC)

We used a slightly modified version of the official method recommended by the European Committee on Antimicrobial Susceptibility Testing (EUCAST, 2024). *V. harveyi* mutant strain MM30 had been cultured overnight in medium at 30 °C in the Environmental Shaker-Incubator: ES-20 (Biosan, Riga. Latvia) and was diluted with the same medium to a density of 0.1 McFarland units. Subsequently, this diluted suspension was dispensed into a 96-well microtiter plate. The test compounds, dissolved in DMSO to a concentration of 1.28 mg/mL, were introduced to the bacterial culture in the first row of wells, resulting in a final concentration of 128 μg/mL. In each subsequent row, the concentration was halved, continuing this serial dilution process until a concentration of 2 μg/mL was reached. The last row of wells served as a control of growth and contained the bacterial suspension only. The plate was then incubated for 24 h at a temperature of 30 °C. After incubation, the absorbance of the samples was measured (at λ = 600 nm), to assess the effects of the test compounds on bacterial growth and viability.

The MRSA strain 7112 was cultured overnight at 37 °C on a solid medium containing agar and Mueller–Hinton broth. A small portion of bacteria was then scraped from the Petri dish and diluted with liquid Mueller–Hinton (MH) medium to achieve a density of 0.5 McFarland units. Next, pure sterile MH medium was added to a 96-well microtiter plate. The test compounds, dissolved in DMSO, were added to the wells and diluted using the same procedure as described for *V. harveyi*. The plate was then inoculated with the diluted culture of MRSA 7112 and incubated at 37 °C for 24 h. Following incubation, the absorbance was measured analogously to the procedure used for *V. harveyi*. To ensure the accuracy and reliability of the results, the experiment was conducted in triplicate, incorporating both a positive control (**17**) and a negative control (DMSO).

### 2.5. Inhibition of AI-2-Dependent QS in V. harveyi MM30

The experimental protocol followed the methodology outlined by Ramić et al. (Ramić, 2022). In this experiment, we initially cultured the MRSA 7112 strain overnight at 37 °C in MH medium. The bacterial culture was then centrifuged, and the supernatant was filtered using a 0.2 μm microfilter syringe to remove all bacterial cells, leaving behind a medium rich in AI-2. The AI-2-rich medium was then added at a ratio of 1 part of AI-2 medium to 8.5 parts of AB medium containing an overnight culture of *V. harveyi* MM30 (grown at 30 °C, diluted to a density of 0.1 McFarland units). A total of 9.5 millilitres of the mixture was thoroughly shaken and then transferred to a microtiter plate with white walls and a transparent bottom. The test compounds were added to the plate and subsequently diluted, using the same method employed in the MIC experiments. The highest concentration used was half of the MIC. The total volume of the experimental mixture dispensed into each well was standardized to 100 µL. The thermostat of the reader was set to 30 °C, to maintain consistent temperature conditions throughout the experiment. Absorbance (at λ = 600 nm) and luminescence were measured every 30 min for 24 h with spectrophotometric reader FLUOstar Omega (BMG Labtech).

The assay was repeated independently, four times. The results are expressed as the ratio of luminescence to absorbance, each compared to the negative control (DMSO). Chlorogenic acid (**19**) was used as a positive control. GraphPad Prism 10 was used to calculate standard errors of the mean, while IBM SPSS Statistics 26.0 was employed to evaluate the significance of the results.

### 2.6. Inhibition of AI-2 Independent QS in V. harveyi MM30

The method for this assay was identical to that of the previous experiment, except that the AI-2-rich medium was not included in this experiment and *V. harveyi* MM30 was incubated in pure AB medium. instead.

### 2.7. Inhibition of AI-2 Production in MRSA 7112

The MRSA 7112 culture in liquid MH medium, initially at a concentration of 1 McFarland unit, was diluted 100× with a pure MH medium. Next, solutions of the test compounds in DMSO were added to achieve a range of subinhibitory concentrations. Following 24 h incubation, the bacterial culture was transferred to 2 mL Eppendorf tubes and centrifuged for 2 min at 10,800 rpm. Then, 10 μL of the resulting supernatant from each tube was combined with 190 μL of *V. harveyi* MM30 culture (AB medium) in a microtitration plate with white walls. Absorbance and bioluminescence measurements were conducted using the same method as in the previous experiments.

### 2.8. Statistical Analysis

Statistical analyses were performed using IBM SPSS Statistics for Windows, version 26.0 (Armonk, NY, USA). Data are presented as the mean ± standard error of the mean (SEM). Graphs were generated, and SEM was calculated using GraphPad Prism software, version 10. Group comparisons were conducted using the Mann–Whitney U test.

## 3. Results and Discussion

### 3.1. Determination of MIC

The antibacterial activity of test compounds **1**–**17** was tested against both *V. harveyi* MM30 and MRSA 7112. All of the test compounds exhibited much greater activity against Gram-positive MRSA than against Gram-negative *V. harveyi*. This more pronounced effect on Gram-positive bacteria has been well reported for both cannabinoids [32] and arylbenzofurans (Naik, 2015). The higher activity against various MRSA strains reported by Appendino et al. [33] for neutral (**1**,**2**,**3**) and acidic cannabinoids (**4**,**5**,**6**) may be attributed to the use of different, potentially more susceptible, strains, compared to those used in our study. The obtained values of MIC are summarized in the Table 1.

Compounds **18**–**20**, considered as potential positive controls for the bioluminescence assay, were tested exclusively for their antibacterial activity against *V. harveyi* MM30. Doxycycline (**17**) was employed as a positive control, with an MIC of 8 µg/mL. Among the test compounds, compound **20** exhibited potent activity at 16 µg/mL, while **19** showed moderate activity at 128 µg/mL. Even though the highest concentration (128 µg/mL) of **9**, **11**, and **13** did cause a decrease in absorbance (by 67%, 67%, and 71%, respectively), noticeable turbidity remained in the wells. The remaining test compounds exhibited no antibacterial effect on *V. harveyi* MM30 (Table 1).

### 3.2. Inhibition of AI-2-Dependent QS in V. harveyi MM30

**Figure 2:**
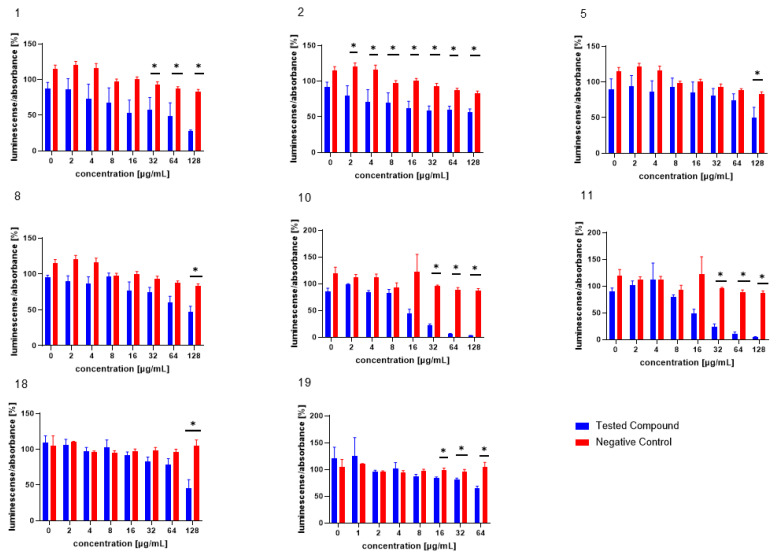
The test compounds **1**, **2**, **5**, **8**, **10**, **11**, **18**, and **19,** which were active in the AI-2-dependent QS assay. DMSO was used as the solvent and was added as the negative control (red columns). The results are expressed as the mean ± SEM for four independent experiments, and are statistically compared to NC (* *p* < 0.05) using the Mann–Whitney U test.

The test compounds were analysed in an assay evaluating the level of bioluminescence in *V. harveyi* MM30 after MRSA filtrate (AI-2) application [28]. The most pronounced dose-dependent ability to inhibit the luminescence of *V. harveyi* MM30 was observed for the compounds **1**, **2**, **10**, and **11** (Figure 2). Statistically significant inhibition was also observed for the compounds **5** and **8** at the highest concentration (128 µg/mL).

Of the compounds we tested as possible positive controls for bioluminescence assays (both with and without the addition of AI-2-containing medium) in *V. harveyi* MM30, **20** was deemed unsuitable for further testing, due to its pronounced antibacterial effect. Compounds **18** and **19** were subsequently evaluated in bioluminescence assays at concentrations ranging from 128 to 2 µg/mL and 64 to 1 µg/mL, respectively. In an assay with AI-2 supplementation, compound **18** significantly decreased luminescence by 54% ± 12% at the highest test concentration (128 µg/mL). Compound **19** exhibited significant activity at concentrations of 64, 32, and 16 µg/mL, reducing bioluminescence by 35% ± 4%, 19% ± 4%, and 16% ± 2%, respectively.

By comparing the molecules efficient in this assay with structurally similar but less-active counterparts, we inferred potential structure–activity relationships. In our study, we observed that neutral cannabinoids exhibited greater activity in reducing AI-2-controlled bioluminescence compared to their acidic forms. The enhanced activity of neutral cannabinoids could be attributed to their greater lipophilicity. Appendino et al. described how structural modifications of cannabinoids that increase the hydrophilicity of the molecule, such as dihydroxylation of the ω-double bond in cannabigerol (CBG), can negatively impact their antibacterial activity [32]. To date, the effect of such structural modifications of cannabinoids on anti-quorum-sensing activity has not been reported.

The importance of maintaining the correct pH level in AI-2 bioluminescence assays is well documented. For example, an acidic environment is known to decrease light production in *V. harveyi*, potentially leading to false positive results [34]. Also, the cannabinoid acids are only weakly acidic, and in the concentrations used probably would not be able to significantly change the pH level of the mixture. Therefore, this factor likely does not account for the observed lower activity of cannabinoid acids. Another factor that could explain the reduced activity of acidic cannabinoids is their lower stability in aqueous environments and at higher temperatures [35]. Although they commonly convert to their neutral forms under these conditions, the resulting concentration of the active neutral product would be much lower than if the corresponding neutral cannabinoid were initially present.

The antibacterial activity of arylbenzofurans from *Morus alba* has been studied extensively and confirmed [36,37]. In our study, we report for the first time the anti-QS activity of arylbenzofurans. This finding is not entirely surprising, as arylbenzofurans contain the furan moiety, which is also present in several well-known QS inhibitors, such as furanocoumarins from *Citrus* sp. [15] and halogenated furanones from the marine algae *Delisea pulchra* [38]. The furan moiety is also present in both AI-2 and AI-1 of *V. harveyi*. Interestingly, the introduction of prenyl groups, which increases lipophilicity, resulted in a significant decrease in activity. Among the arylbenzofurans, the non-prenylated compound **10** exhibited the highest activity. The second most active was compound **11**. Despite being prenylated, it has a significantly lower molecular weight compared to the other test compounds in this structural class. This observation, along with the superior activity of neutral cannabinoids, suggests that the smaller molecular size of those compounds may contribute to their enhanced bioactivity. Among the compounds evaluated as potential positive controls for this assay, compound **19** demonstrated the highest efficiency. This observation aligns with the existing literature on the effects of compound **19** on various bacterial species, such as *P. aeruginosa* [26]. Due to its relatively high efficacy in reducing AI-2-mediated bioluminescence and the low standard error of the mean observed in the experimental results, this substance serves as an appropriate positive control for the assay.

### 3.3. Inhibition of AI-2-Independent QS in V. harveyi MM30

**Figure 3:**
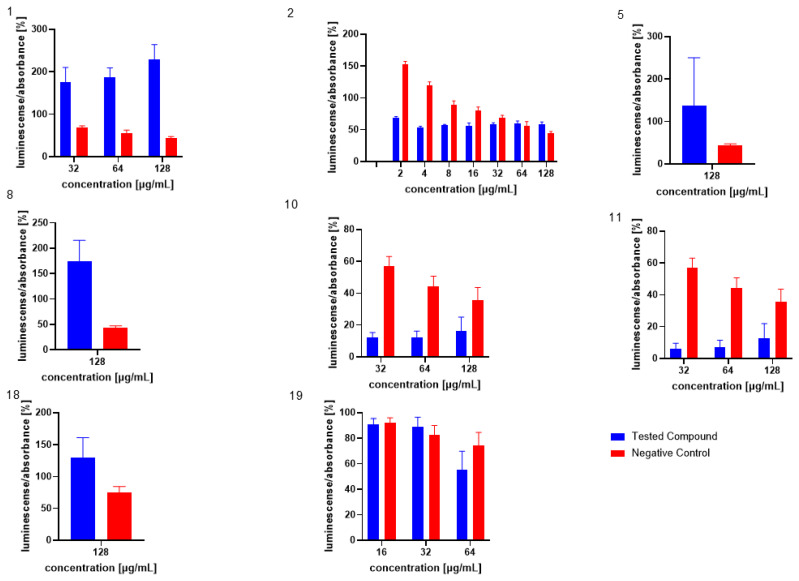
Evaluation of test compounds in the AI-2-independent bioassay. None of the test compounds exhibited significant activity in this assay. For comparison, the activity of compounds and concentrations previously identified as active in the AI-2-dependent quorum-sensing (QS) assay is presented. Dimethyl sulfoxide (DMSO) served as the solvent control, and was included as the negative control (red bars). Data are expressed as the mean ± SEM from four independent experiments, and were statistically analysed, relative to the negative control, using the Mann–Whitney U test.

**Figure 4:**
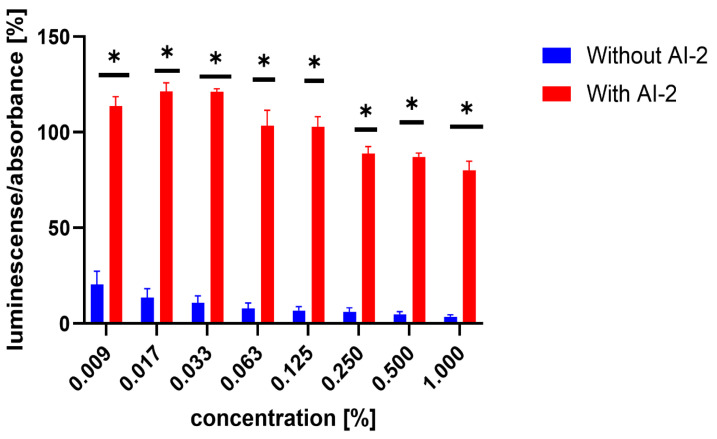
Comparison of luminescence-to-absorbance ratios obtained from negative controls (DMSO) in AI-2-dependent and AI-2-independent quorum-sensing (QS) assays. The x-axis represents the percentage concentrations of DMSO in the samples (percentage of DMSO in the inoculated AB medium), consistent with the concentrations used to dissolve the test compounds. Data are presented as the mean ± SEM from four independent experiments, and were statistically analysed, relative to the negative control, using the Mann–Whitney U test (* *p* < 0.05).

Similarly, the influence of the test compounds was evaluated, to analyse the potential decrease in the bioluminescence of *V. harveyi* without the application of MRSA 7112 filtrate (AI-2). In this assay, none of the test compounds significantly decreased the bioluminescence of *V. harveyi* MM30 compared to the negative control (Figure 3), and compounds **7** and **8** showed an apparent positive effect on bioluminescence. Substance **19** (the positive control in the previous assay) did not significantly reduce light production in the absence of AI-2 enriched medium. Although it caused an average decrease of 44% in bioluminescence, the large standard error of the mean (±14%) indicated considerable variability in the results. The method used to evaluate non-AI-2-controlled bioluminescence had significant limitations. Given that bioluminescence in *V. harveyi* MM30 is primarily regulated by the AI-2/*lux-S* system [12], the observed luminescence values in this experiment were lower in magnitude and exhibited greater variability (Figure 4). This led to larger standard errors of the mean compared to experiments conducted in a medium enriched with AI-2. Additionally, the influence of the solvent used (DMSO) was found to be non-negligible for most of the concentrations. These factors could contribute to the apparent induction of luminescence in some of the compounds, although other causes, including the actual ability of our compounds to support bioluminescence (and quorum sensing), cannot be ruled out. Several natural compounds are known to increase quorum sensing in various strains of bacteria. For instance, Ahmad et al. investigated this phenomenon in natural monoterpenoids [39]. Their findings highlighted the significant role of stereochemistry in QS modulation. Specifically, the (+)- enantiomers of carvone, limonene, and borneol were observed to enhance (QS-regulated) violacein production in *Chromobacterium violaceum* and pyocyanin production in *P. aeruginosa*. Conversely, their laevorotary analogues exhibited inhibitory effects on its production.

### 3.4. Inhibition of AI-2 Production in MRSA 7112

**Figure 5:**
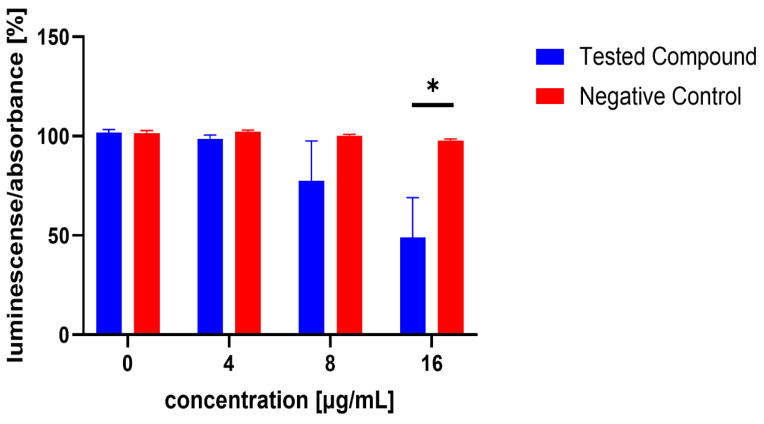
Effect of test compound **5** on AI-2 production in *Staphylococcus aureus* MRSA 7112. DMSO was used as the solvent and served as the negative control, represented by red bars. Results are presented as the mean ± SEM from four independent experiments, and were compared to the negative control using the Mann–Whitney U test (* *p* < 0.05).

**Figure 6:**
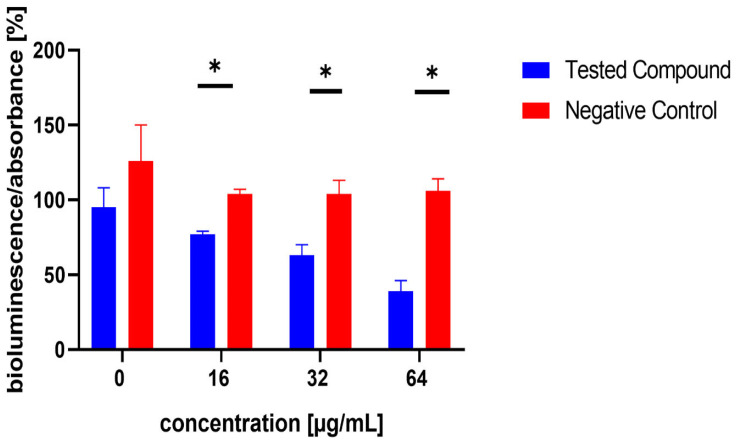
Effect of test compound **10** on AI-2 production in *Staphylococcus aureus* MRSA 7112. DMSO, utilized as the solvent, also functioned as the negative control, shown as red bars in the figure. Data are shown as mean ± SEM from four independent experiments and were statistically evaluated against the negative control, using the Mann–Whitney U test (* *p* < 0.05).

**Figure 7:**
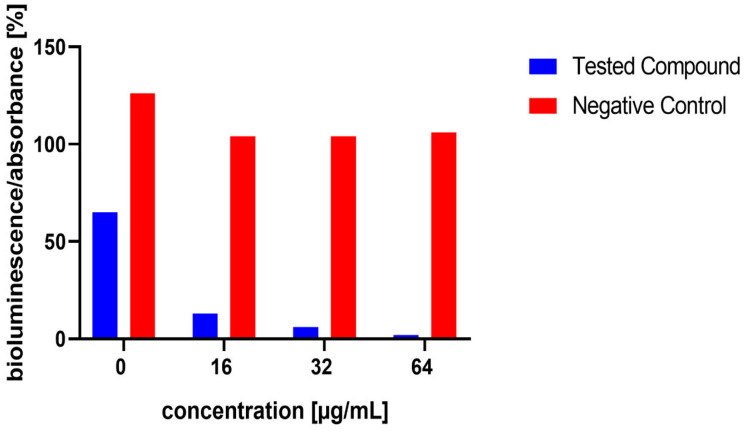
Effect of test compound **11** on AI-2 Production in *Staphylococcus aureus* MRSA 7112. The results are considered not significant, due to the potential interference from the antibacterial activity of the compound. DMSO acted as the negative control, indicated by red bars. Data are expressed as the mean ± SEM from four independent replicates and analysed for statistical significance in comparison to the negative control, using the Mann–Whitney U test.

The influence of the compounds **1**, **2**, **5**, **10**, and **11** on the production of AI-2 was tested in MRSA 7112. Among the compounds tested, **10** exhibited a significant reduction in bioluminescence across all concentrations tested (23% ± 2%, 37% ± 7%, 61% ± 7% for 16, 32, and 64 µg/mL, respectively), while **5** demonstrated a significant decrease only at the highest concentration used (64 µg/mL), resulting in a mean reduction of bioluminescence by −51% ± 20%. Although compound **11** also significantly decreased bioluminescence at all tested concentrations, this effect was likely attributable to the pronounced antibacterial activity observed in the assay, as MRSA cultures treated with this compound showed no visible turbidity, suggesting substantial inhibition of bacterial growth. The results are shown in Figure 5, Figure 6 and Figure 7.

The main limitation of the method we employed to evaluate AI-2 production in MRSA is its inability to correlate AI-2 production with the count of viable bacterial cells in the MRSA culture. Despite establishing MIC values for **1**, **2**, and **5** for MRSA in microtitration plate tests and using sub-MIC concentrations (1/4, 1/2, and 1/8 of their MICs, respectively), some cultures did not exhibit noticeable turbidity. However, this variability was not consistent across all repetitions of the experiment; for instance, in the case of 16 µg/mL of compound **1**, turbidity ranged from 0 to 0.5 McFarland units. This inconsistency contributed to significant variations and higher standard errors of the mean, especially at the highest concentration (16 µg/mL). Most importantly, due to this limitation, the potential influence of bacteriostatic effects on MRSA cannot be disregarded.

## 4. Conclusions

Our study demonstrates the specific anti-quorum-sensing activity of natural phenolic compounds from *Cannabis sativa* and *Morus alba*, using *Vibrio harveyi* MM30 as a biosensor reporter strain. Cannabigerol (**1**), cannabidiol (**2**), moracin M (**10**), and moracin C (**11**) were identified as potent inhibitors of AI-2-mediated quorum-sensing-controlled bioluminescence. Chlorogenic acid (**19**) was validated as a positive control, due to its consistent and significant luminescence reduction at sub-MIC concentrations.

These findings build upon earlier reports of quorum-sensing inhibition by cannabinoids, specifically cannabigerol [12], by reinforcing its activity and highlighting cannabidiol as an additional potent inhibitor. This expands the understanding of neutral cannabinoids’ roles in quorum-sensing interference. The identification of moracin M and moracin C further broadens the spectrum of phenolic compounds with activity against AI-2-mediated communication, aligning with prior studies suggesting the quorum-sensing inhibitory potential of molecules with furan moiety.

In MRSA 7112, moracin M and cannabigerolic acid (**5**) significantly affected AI-2 production, consistent with the known influence of plant-derived phenolics on quorum-sensing systems in Gram-positive pathogens [40]. However, the potential contribution of antibacterial effects cannot be ruled out, underscoring the need for further investigation to disentangle these effects from direct quorum-sensing inhibition.

Further bioluminescence assays without AI-2-containing medium did not identify significant inhibitors of non-AI-2-controlled quorum-sensing pathways. However, the observed stimulation of bioluminescence by cannabinolic acid (**7**) and cannflavin B (**8**) at high concentrations suggests a pathway-specific response that warrants additional study.

Overall, our findings support the growing evidence of plant-derived phenolic compounds as modulators of bacterial communication, with implications for developing quorum-sensing inhibitors to combat antimicrobial resistance. The future application of natural compounds with QS inhibitory properties offers promising avenues for combating bacterial infections, particularly in an era of rising antibiotic resistance. Cannabinoids, such as cannabidiol and cannabigerol, hold significant potential in antimicrobial therapy. Their ability to inhibit biofilm formation and disrupt QS-regulated virulence factors suggests their use as adjuvants in treating chronic infections, particularly those involving *P. aeruginosa* or methicillin-resistant *S. aureus* (MRSA). Future formulations could include cannabinoids in topical treatments for wound infections or as coatings for medical devices to prevent biofilm-related complications. Flavonoids like naringenin and quercetin may be integrated into dietary supplements or functional foods to help mitigate infections in vulnerable populations. Their QS-inhibitory effects could also be harnessed in agriculture to protect crops from bacterial pathogens, reducing reliance on chemical pesticides. Additionally, flavonoids could be developed into pharmaceutical agents for diseases involving biofilm-associated infections, such as dental caries and catheter-related infections. Arylbenzofurans and related compounds may find use in the food industry, to reduce bacterial contamination or spoilage by targeting QS pathways in pathogens like *Salmonella* or *Listeria*. Similarly, these compounds could enhance the effectiveness of existing preservatives when combined. Beyond clinical and agricultural applications, these compounds could play a role in environmental biotechnology, such as controlling harmful biofilms in water treatment systems or on industrial surfaces. The ability of natural QS inhibitors to disrupt biofilms in non-biological settings offers a safer alternative to traditional biocides. While these applications hold immense promise, future research should focus on enhancing the bioavailability, stability, and specificity of these compounds. Advancements in nanotechnology and drug delivery systems could improve their therapeutic potential, while exploring combinations with existing antibiotics or other natural compounds could yield synergistic effects. Ultimately, these natural QS inhibitors could revolutionize how we manage bacterial infections and related challenges across multiple sectors.

## Figures and Tables

**Table 1:** Antimicrobial activity of test compounds against MRSA 7112 and *V. harveyi* MM30 expressed as MIC (µg/mL).

Class of Compounds	Number	Name	MIC MRSA	MIC *V.h.*
Neutral cannabinoid	1	Cannabigerol	64 µg/mL	>128 µg/mL
	2	Cannabidiol	32 µg/mL	>128 µg/mL
	3	Cannabinol	64 µg/mL	>128 µg/mL
Cannabinoid acid	4	Tetrahydrocannabinolic acid	128 µg/mL	>128 µg/mL
	5	Cannabigerolic acid	128 µg/mL	>128 µg/mL
	6	Cannabidiolic acid	64 µg/mL	>128 µg/mL
	7	Cannabinolic acid	128 µg/mL	>128 µg/mL
Prenylated flavonoid	8	Cannflavin B	64 µg/mL	>128 µg/mL
Non-prenylated flavonoid	9	Quercetin	>128 µg/mL	>128 µg/mL
Non-prenylated arylbenzofuran	10	Moracin M	>128 µg/mL	>128 µg/mL
Prenylated arylbenzofuran	11	Moracin C	128 µg/mL	>128 µg/mL
	12	Moracin T	>128 µg/mL	>128 µg/mL
	13	Albanol B	16 µg/mL	>128 µg/mL
	14	Mulberrofuran Y	128 µg/mL	>128 µg/mL
	15	Mulberrofuran G	>128 µg/mL	>128 µg/mL
	16	Mulberrofuran K	>128 µg/mL	>128 µg/mL
Positive control	17	Doxycycline	4 µg/mL	8 µg/mL
	18	Caffeine	N.D. *	>128 µg/mL
	19	Chlorogenic acid	N.D.	128 µg/mL
	20	Epigallocatechine gallate	N.D.	16 µg/mL

* not determined.

## Data Availability

Data generated in this research are available at the authors.

## Supplementary Materials

The following supporting information can be downloaded at: https://www.mdpi.com/article/10.3390/microorganisms13020287/s1, Figure S1: Extraction process of THCA-rich *C. sativa* flower; Figure S2: Identification of compound **1** (cannabigerol) with UHPLC/MS; Figure S3: Identification of compound **3** (cannabinol) with UHPLC/MS; Figure S4: Identification of compound **4** (tetrahydrocannabinolic acid) with UHPLC/MS; Figure S5: Identification of compound **7** (cannabinolic acid) with UHPLC/MS; Figure S6: Identification of compound **8** (cannflavin B) with UHPLC/MS; Figure S7: Extraction process of CBGA-rich *C. sativa* flower trichomes; Figure S8: Identification of compound **5** (cannabigerolic acid) with UHPLC/MS; Figure S9: Extraction process of CBDA-rich *C. sativa* flower; Figure S10: Identification of compound **6** (cannabidiolic acid) with UHPLC/MS; Table S1: Column chromatography method for the separation of chloroform fraction of tetrahydrocannabinol-rich *C. sativa* extract; Table S2: Column chromatography method for the separation of the chloroform fraction of cannabigerol-rich *C. sativa* extract; Table S3: Flash chromatography method for cannabidiol-rich *C. sativa* extract.

## Abbreviations

The following abbreviations are used in this manuscript:

AB	Autoinducer-2 bioassay
AI-1	Autoinducer-1
AI-2	Autoinducer-2
CAI-1	Cholera autoinducer-1
CBDA	Cannabidiolic acid
CBG	Cannabigerol
CBGA	Cannabigerolic acid
DMSO	Dimethylsulfoxide
MH	Mueller–Hinton (medium)
MIC	Minimal inhibitory concentration
MRSA	Methicilin-resistant Staphylococcus aureus
NO	Nitric oxide
QS	Quorum sensing
QSI	Quorum-sensing inhibitor
THCA	Tetrahydrocannabinolic acid

## References

[1] Schluter J., Schoech A.P., Foster K.R., Mitri S. (2016). The Evolution of Quorum Sensing as a Mechanism to Infer Kinship. PLoS Comput. Biol..

[2] Kolenbrander P.E., Andersen R.N., Blehert D.S., Egland P.G., Foster J.S., Palmer R.J. (2002). Communication among Oral Bacteria. Microbiol. Mol. Biol. Rev..

[3] Fuqua W.C., Winans S.C., Greenberg E.P. (1994). Quorum Sensing in Bacteria: The LuxR-LuxI Family of Cell Density-Responsive Transcriptional Regulators. J. Bacteriol..

[4] Defoirdt T., Sorgeloos P. (2012). Monitoring of *Vibrio harveyi* Quorum Sensing Activity in Real Time during Infection of Brine Shrimp Larvae. ISME J..

[5] Aharoni R., Bronstheyn M., Jabbour A., Zaks B., Srebnik M., Steinberg D. (2008). Oxazaborolidine Derivatives Inducing Autoinducer-2 Signal Transduction in *Vibrio harveyi*. Bioorg. Med. Chem..

[6] Cao J.G., Meighen E.A. (1989). Purification and Structural Identification of an Autoinducer for the Luminescence System of *Vibrio harveyi*. J. Biol. Chem..

[7] Kadirvel M., Fanimarvasti F., Forbes S., McBain A., Gardiner J.M., Brown G.D., Freeman S. (2014). Inhibition of Quorum Sensing and Biofilm Formation in *Vibrio harveyi* by 4-Fluoro-DPD; a Novel Potent Inhibitor of AI-2 Signalling. Chem. Commun..

[8] Higgins D.A., Pomianek M.E., Kraml C.M., Taylor R.K., Semmelhack M.F., Bassler B.L. (2007). The Major Vibrio Cholerae Autoinducer and Its Role in Virulence Factor Production. Nature.

[9] Henares B., Xu Y., Boon E. (2013). A Nitric Oxide-Responsive Quorum Sensing Circuit in *Vibrio harveyi* Regulates Flagella Production and Biofilm Formation. Int. J. Mol. Sci..

[10] Keller L., Surette M.G. (2006). Communication in Bacteria: An Ecological and Evolutionary Perspective. Nat. Rev. Microbiol..

[11] Surette M.G., Miller M.B., Bassler B.L. (1999). Quorum Sensing in *Escherichia coli*, *Salmonella typhimurium*, and *Vibrio harveyi*: A New Family of Genes Responsible for Autoinducer Production. Proc. Natl. Acad. Sci. USA.

[12] Aqawi M., Gallily R., Sionov R.V., Zaks B., Friedman M., Steinberg D. (2020). Cannabigerol Prevents Quorum Sensing and Biofilm Formation of *Vibrio harveyi*. Front. Microbiol..

[13] Kumar L., Chhibber S., Kumar R., Kumar M., Harjai K. (2015). Zingerone Silences Quorum Sensing and Attenuates Virulence of Pseudomonas Aeruginosa. Fitoterapia.

[14] Martín-Rodríguez A.J., Ticona J.C., Jiménez I.A., Flores N., Fernández J.J., Bazzocchi I.L. (2015). Flavonoids from Piper Delineatum Modulate Quorum-Sensing-Regulated Phenotypes in *Vibrio harveyi*. Phytochemistry.

[15] Girennavar B., Cepeda M.L., Soni K.A., Vikram A., Jesudhasan P., Jayaprakasha G.K., Pillai S.D., Patil B.S. (2008). Grapefruit Juice and Its Furocoumarins Inhibits Autoinducer Signaling and Biofilm Formation in Bacteria. Int. J. Food Microbiol..

[16] Vikram A., Jayaprakasha G.K., Jesudhasan P.R., Pillai S.D., Patil B.S. (2010). Suppression of Bacterial Cell–Cell Signalling, Biofilm Formation and Type III Secretion System by Citrus Flavonoids. J. Appl. Microbiol..

[17] Parai D., Banerjee M., Dey P., Chakraborty A., Islam E., Mukherjee S.K. (2018). Effect of Reserpine on *Pseudomonas aeruginosa* Quorum Sensing Mediated Virulence Factors and Biofilm Formation. Biofouling.

[18] Brackman G., Defoirdt T., Miyamoto C., Bossier P., Van Calenbergh S., Nelis H., Coenye T. (2008). Cinnamaldehyde and Cinnamaldehyde Derivatives Reduce Virulence in Vibrio Spp. by Decreasing the DNA-Binding Activity of the Quorum Sensing Response Regulator LuxR. BMC Microbiol..

[19] Paczkowski J.E., Mukherjee S., McCready A.R., Cong J.-P., Aquino C.J., Kim H., Henke B.R., Smith C.D., Bassler B.L. (2017). Flavonoids Suppress Pseudomonas Aeruginosa Virulence through Allosteric Inhibition of Quorum-Sensing Receptors. J. Biol. Chem..

[20] Soni D., Smoum R., Breuer A., Mechoulam R., Steinberg D. (2015). Effect of the Synthetic Cannabinoid HU-210 on Quorum Sensing and on the Production of Quorum Sensing-Mediated Virulence Factors by *Vibrio harveyi*. BMC Microbiol..

[21] Friedman L., Smoum R., Feldman M., Mechoulam R., Steinberg D. (2019). Does the Endocannabinoid Anandamide Affect Bacterial Quorum Sensing, Vitality, and Motility?. Cannabis. Cannabinoid. Res..

[22] Martinelli D., Grossmann G., Séquin U., Brandl H., Bachofen R. (2004). Effects of Natural and Chemically Synthesized Furanones on Quorum Sensing in Chromobacterium Violaceum. BMC Microbiol..

[23] Laganenka L., Sander T., Lagonenko A., Chen Y., Link H., Sourjik V. (2019). Quorum Sensing and Metabolic State of the Host Control Lysogeny-Lysis Switch of Bacteriophage T1. mBio.

[24] Norizan S., Yin W.-F., Chan K.-G. (2013). Caffeine as a Potential Quorum Sensing Inhibitor. Sensors.

[25] Castillo S., Heredia N., García S. (2015). 2(5H)-Furanone, Epigallocatechin Gallate, and a Citric-Based Disinfectant Disturb Quorum-Sensing Activity and Reduce Motility and Biofilm Formation of Campylobacter Jejuni. Folia Microbiol..

[26] Xu W., Zhang X., Wang L., Zeng W., Sun Y., Zhou C., Zhou T., Shen M. (2022). Effect of Chlorogenic Acid on the Quorum-sensing System of Clinically Isolated Multidrug-resistant *Pseudomonas aeruginosa*. J. Appl. Microbiol..

[27] Čulenová M., Sychrová A., Hassan S.T.S., Berchová-Bímová K., Svobodová P., Helclová A., Michnová H., Hošek J., Vasilev H., Suchý P. (2020). Multiple In Vitro Biological Effects of Phenolic Compounds from Morus Alba Root Bark. J. Ethnopharmacol..

[28] Ramić D., Klančnik A., Možina S.S., Dogsa I. (2022). Elucidation of the AI-2 Communication System in the Food-Borne Pathogen Campylobacter Jejuni by Whole-Cell-Based Biosensor Quantification. Biosens. Bioelectron..

[29] Hong N.T.X., Baruah K., Vanrompay D., Bossier P. (2016). Characterization of Phenotype Variations of Luminescent and Non-luminescent Variants of *Vibrio harveyi* Wild Type and Quorum Sensing Mutants. J. Fish Dis..

[30] Mok K.C. (2003). *Vibrio harveyi* Quorum Sensing: A Coincidence Detector for Two Autoinducers Controls Gene Expression. EMBO J..

[31] Cluzel M.-E., Zanella-Cléon I., Cozzone A.J., Fütterer K., Duclos B., Molle V. (2010). The Staphylococcus Aureus Autoinducer-2 Synthase LuxS Is Regulated by Ser/Thr Phosphorylation. J. Bacteriol..

[32] Appendino G., Giana A., Gibbons S., Maffei M., Gnavi G., Grassi G., Sterner O. (2008). A Polar Cannabinoid from *Cannabis sativa* Var. Carma. Nat. Prod. Commun..

[33] Appendino G., Gibbons S., Giana A., Pagani A., Grassi G., Stavri M., Smith E., Rahman M.M. (2008). Antibacterial Cannabinoids from *Cannabis sativa*: A Structure−Activity Study. J. Nat. Prod..

[34] DeKeersmaecker S.C.J., Vanderleyden J. (2003). Constraints on Detection of Autoinducer-2 (AI-2) Signalling Molecules Using *Vibrio harveyi* as a Reporter. Microbiology.

[35] Filer C.N. (2022). Acidic Cannabinoid Decarboxylation. Cannabis. Cannabinoid. Res..

[36] Naik R., Harmalkar D.S., Xu X., Jang K., Lee K. (2015). Bioactive Benzofuran Derivatives: Moracins A–Z in Medicinal Chemistry. Eur. J. Med. Chem..

[37] Škovranová G., Čulenová M., Treml J., Dzurická L., Marova I., Sychrová A. (2022). Prenylated Phenolics from Morus Alba against MRSA Infections as a Strategy for Wound Healing. Front. Pharmacol..

[38] Rasmussen T.B., Manefield M., Andersen J.B., Eberl L., Anthoni U., Christophersen C., Steinberg P., Kjelleberg S., Givskov M. (2000). How Delisea Pulchra Furanones Affect Quorum Sensing and Swarming Motility in Serratia Liquefaciens MG1. Microbiology.

[39] Ahmad A., Viljoen A.M., Chenia H.Y. (2015). The Impact of Plant Volatiles on Bacterial Quorum Sensing. Lett. Appl. Microbiol..

[40] Yue J., Yang H., Liu S., Song F., Guo J., Huang C. (2018). Influence of Naringenin on the Biofilm Formation of Streptococcus Mutans. J. Dent..

