# Supplementary material for: Natural Phenolics Disrupt Microbial Communication by Inhibiting Quorum Sensing
Source: Microorganisms. 2025 Jan 27;13(2):287. doi: 10.3390/microorganisms13020287 (PMC11857621; doi:10.3390/microorganisms13020287)
Supplement: Supplementary file 1 [file microorganisms-13-00287-s001.zip › microorganisms-3443394-supplementary.pdf]

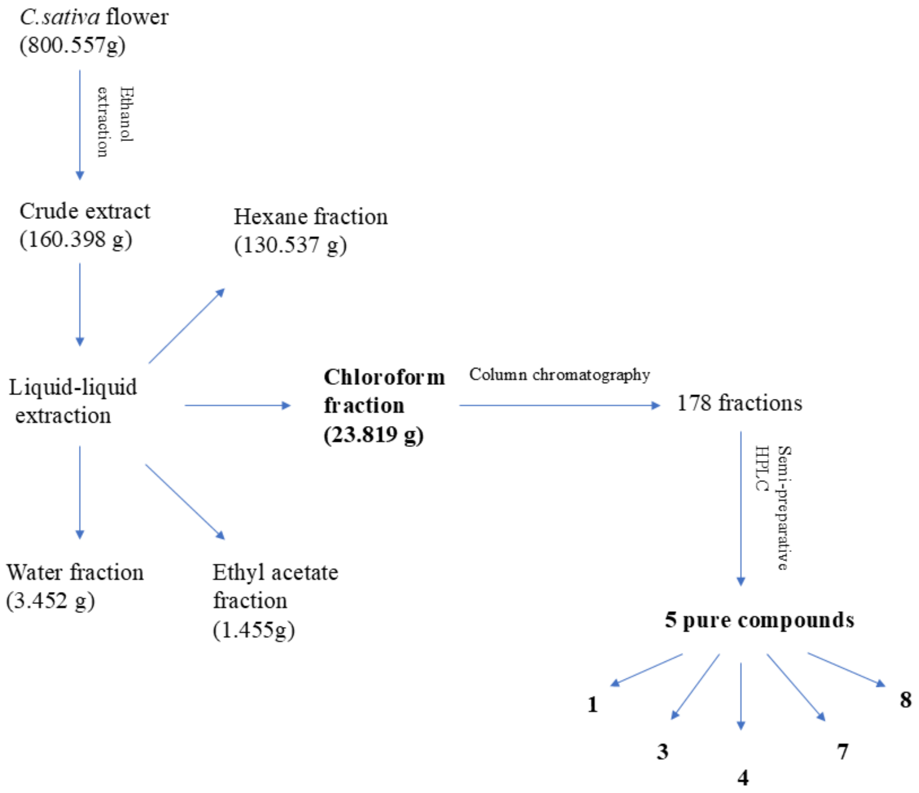

Figure S1. Extraction process of THCA-rich *C. sativa* flower.

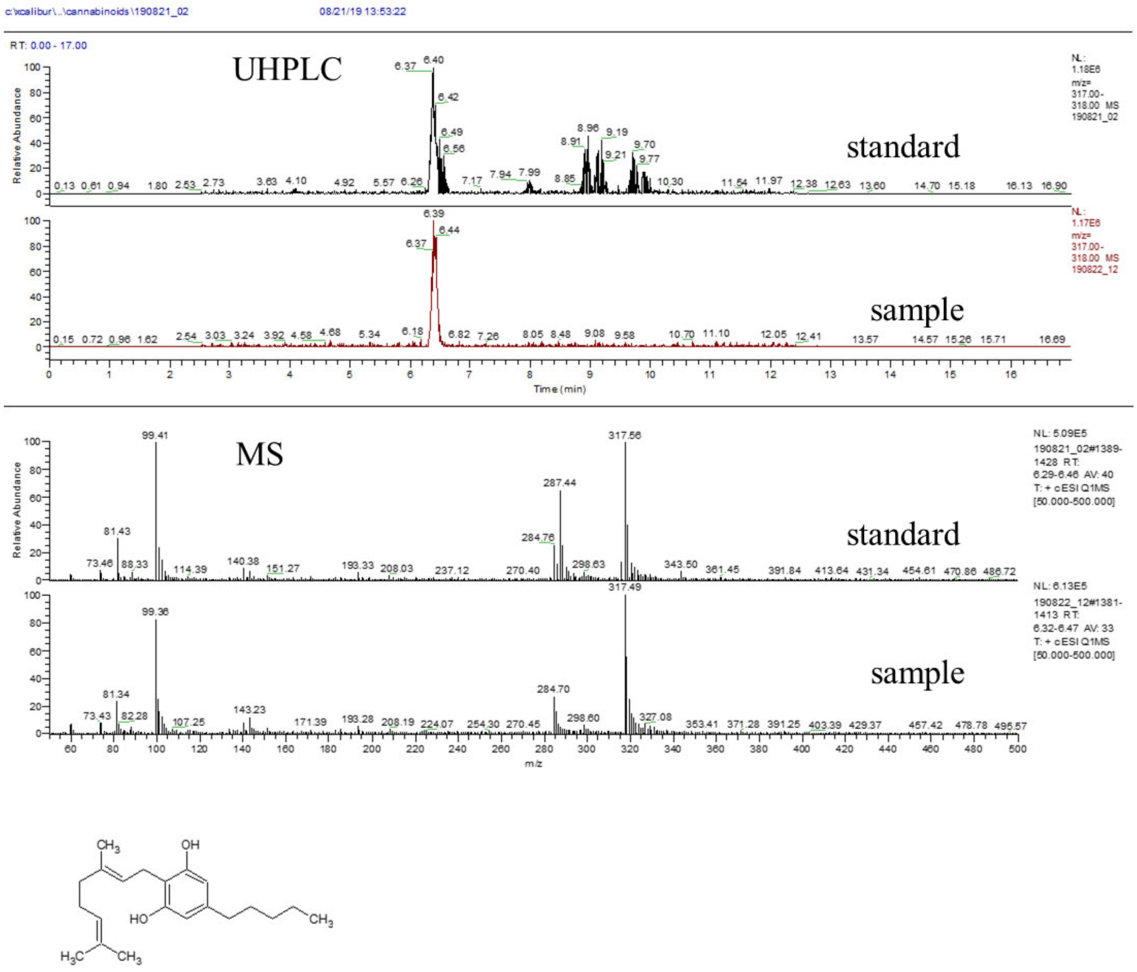

Figure S2. Identification of compound 1 (cannabigerol) with UHPLC/MS.

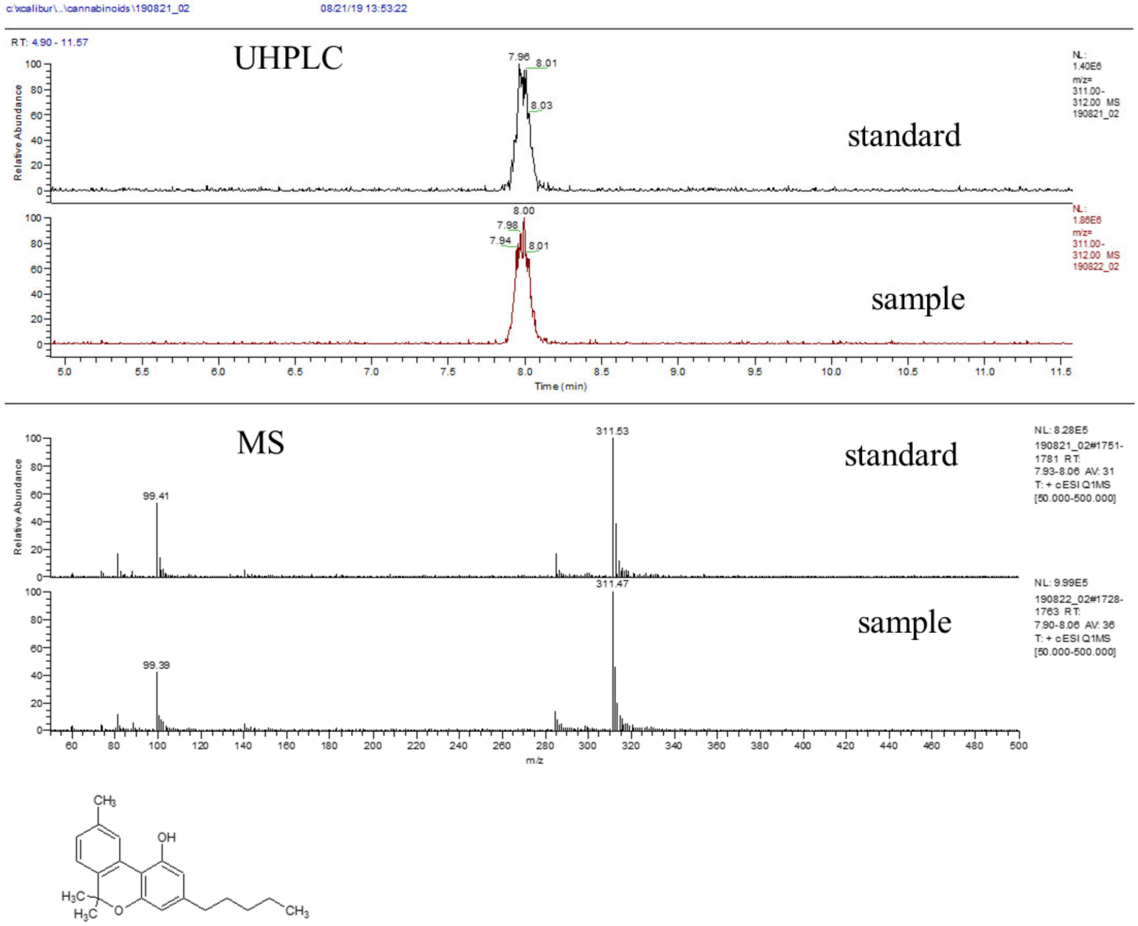

Figure S3. Identification of compound 3 (cannabinol) with UHPLC/MS**.**

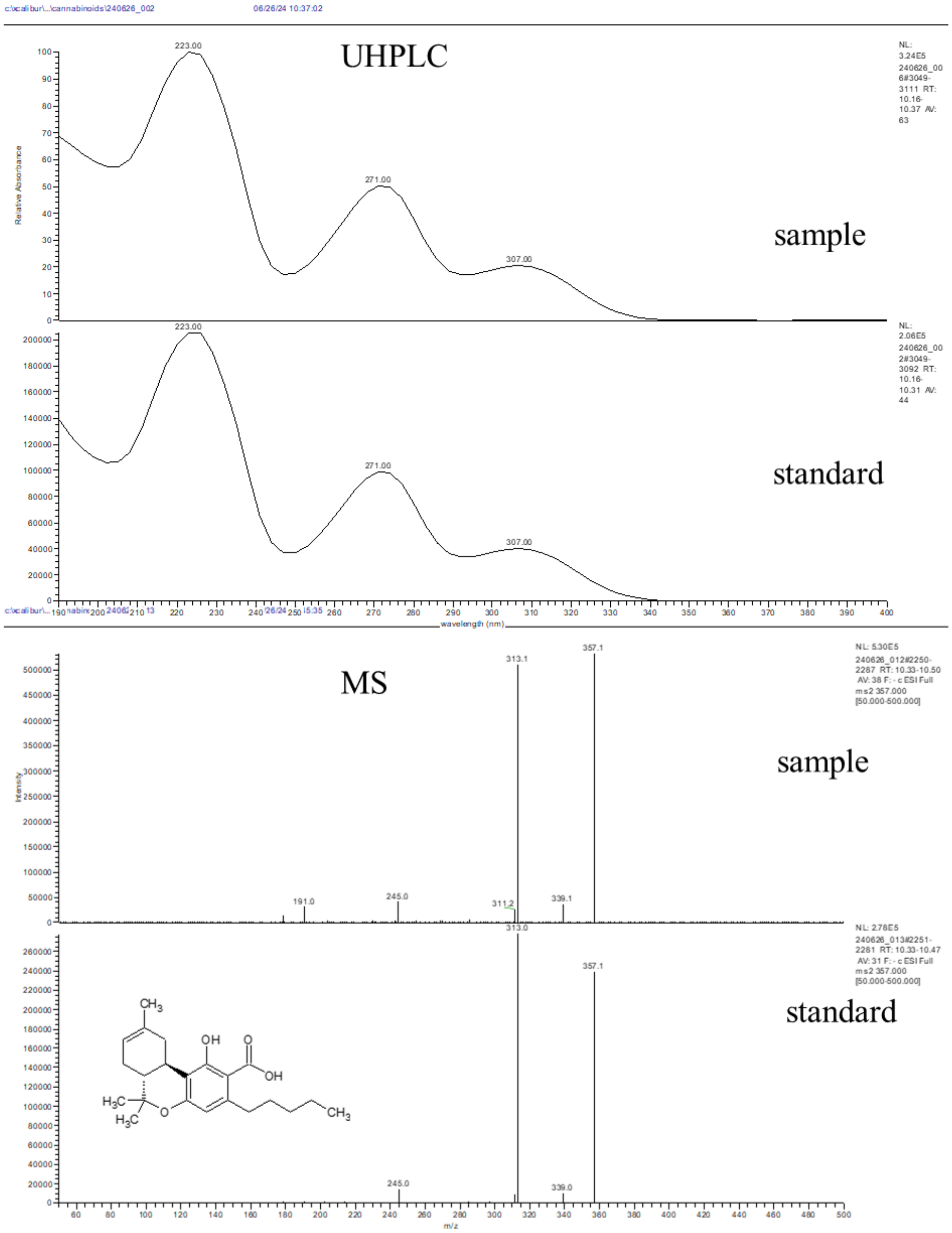

Figure S4. Identification of compound 4 (tetrahydrocannabinolic acid) with UHPLC/MS.

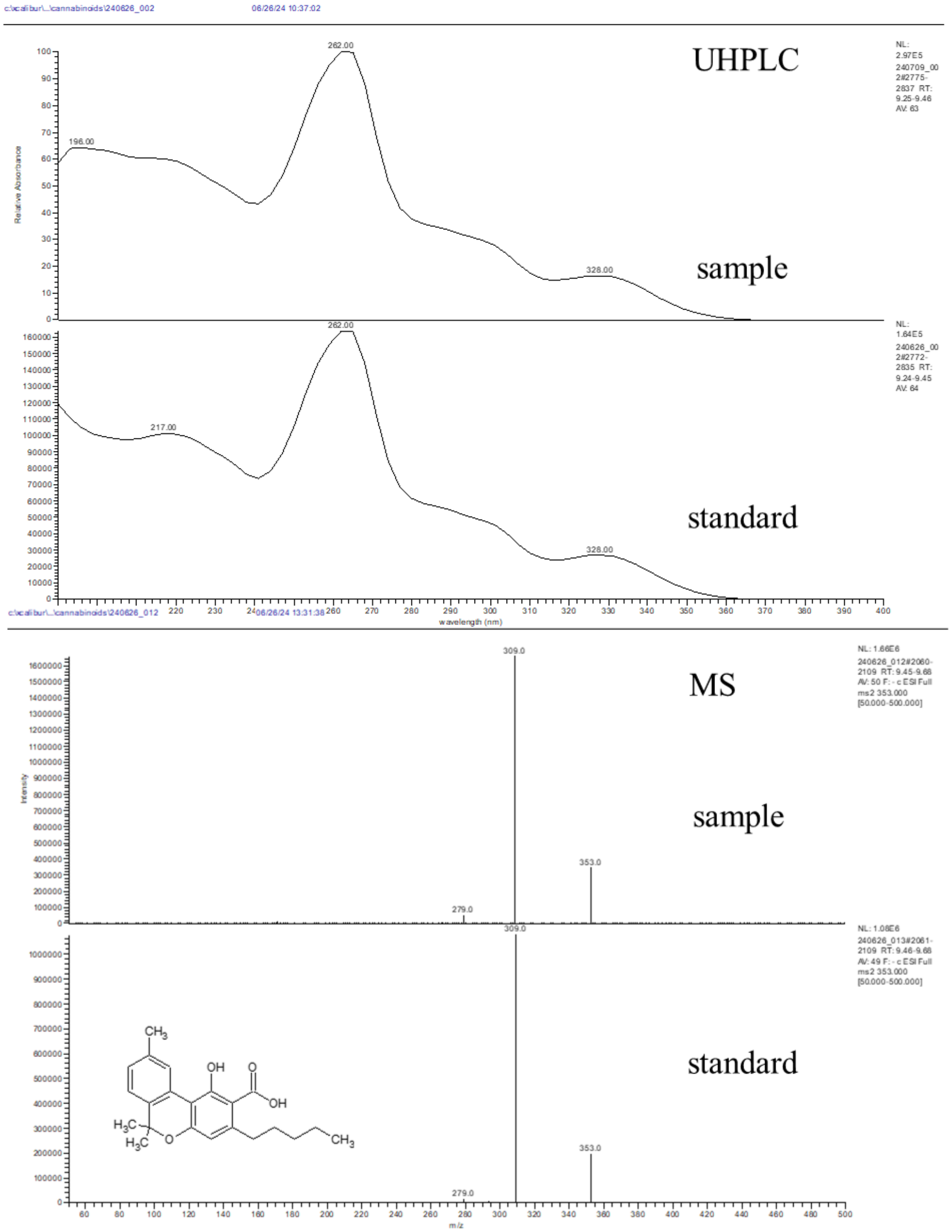

Figure S5. Identification of compound 7 (cannabinolic acid) with UHPLC/MS.

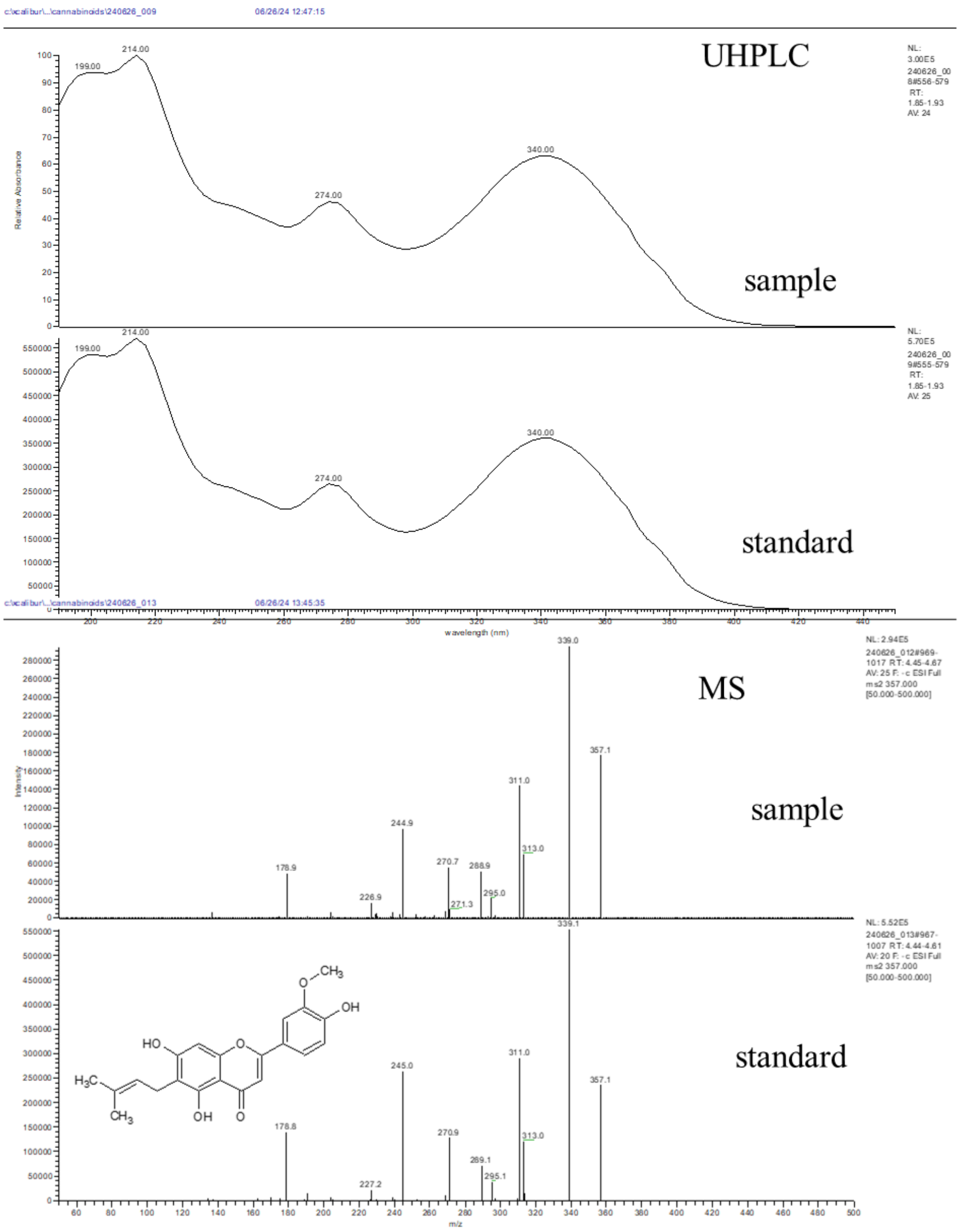

Figure S6. Identification of compound 8 (cannflavin B) with UHPLC/MS.

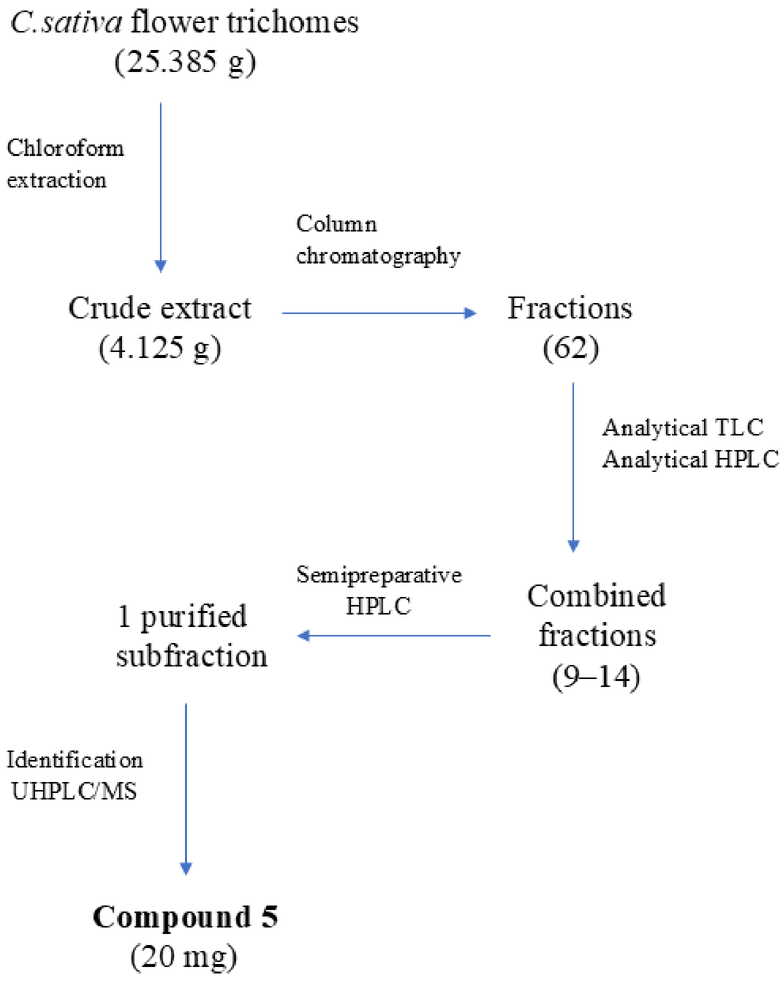

Figure S7. Extraction process of CBGA-rich *C. sativa* flower trichomes.

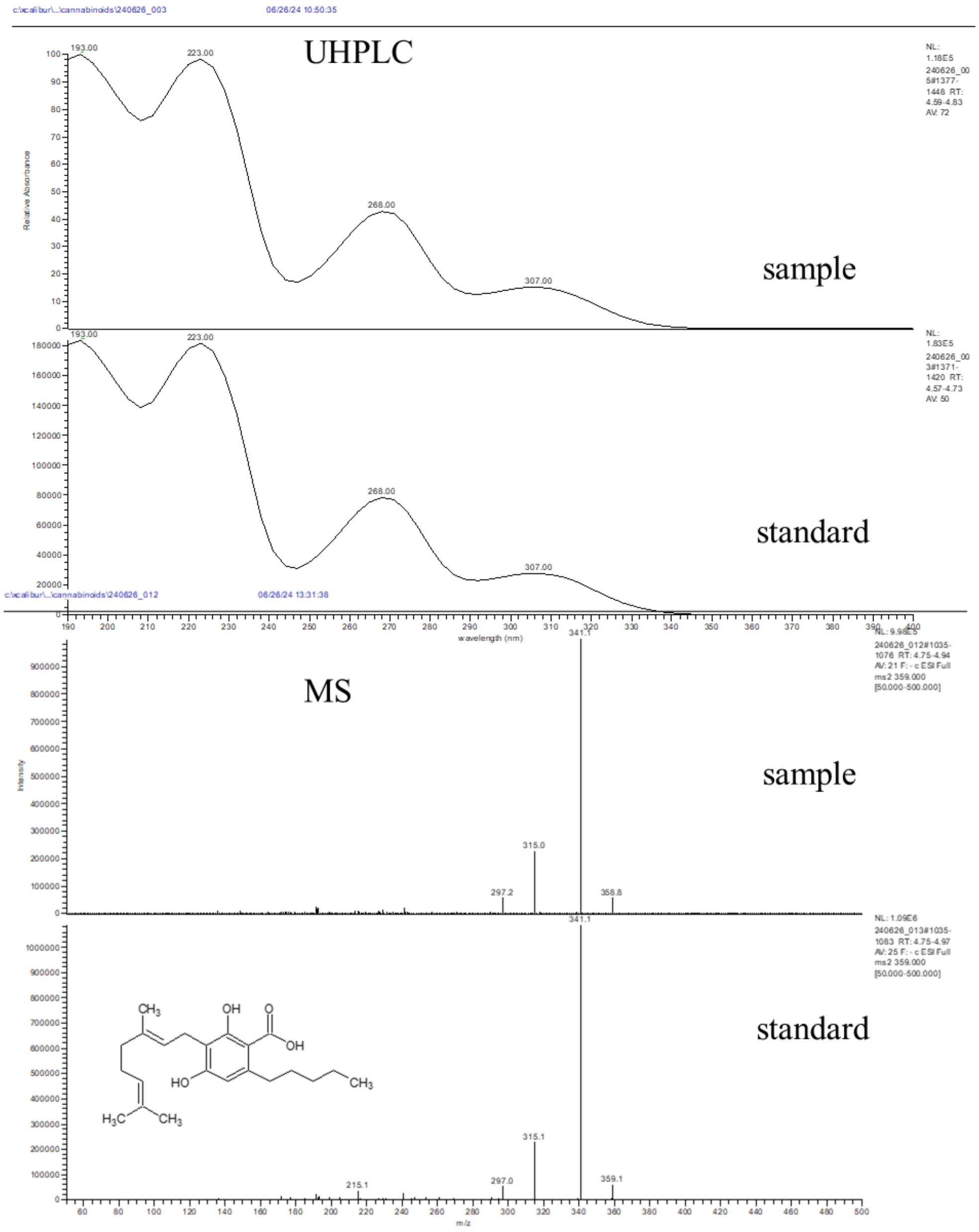

Figure S8. Identification of compound 5 (cannabigerolic acid) with UHPLC/MS.

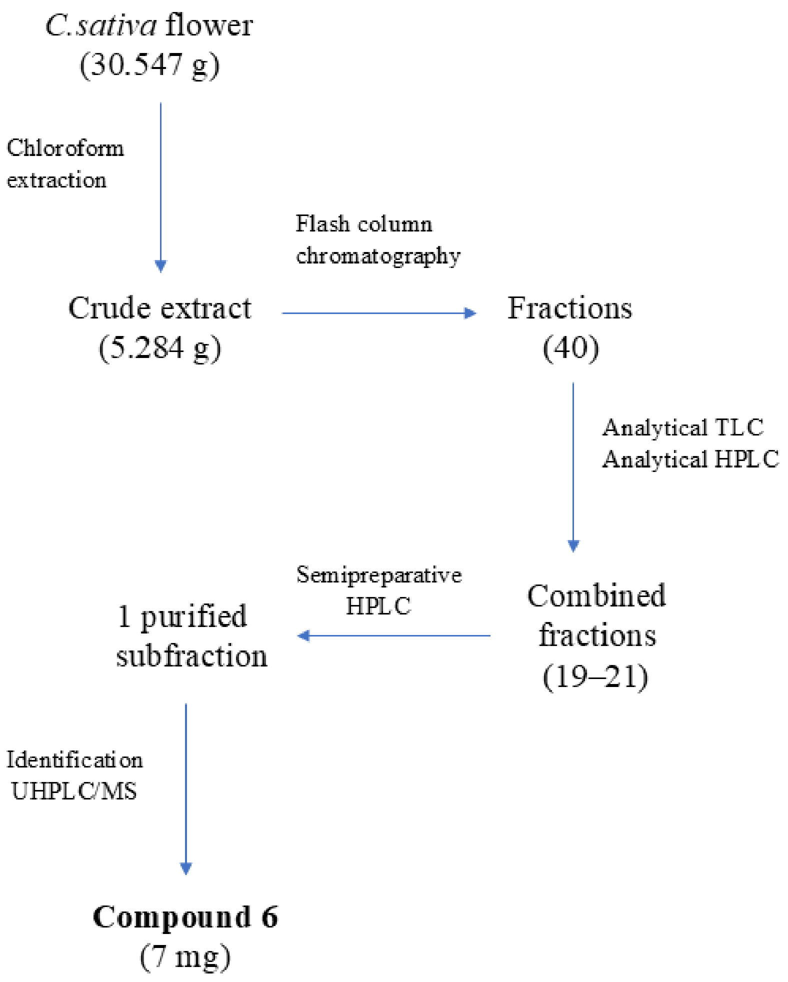

Figure S9. Extraction process of CBDA-rich *C. sativa* flower.

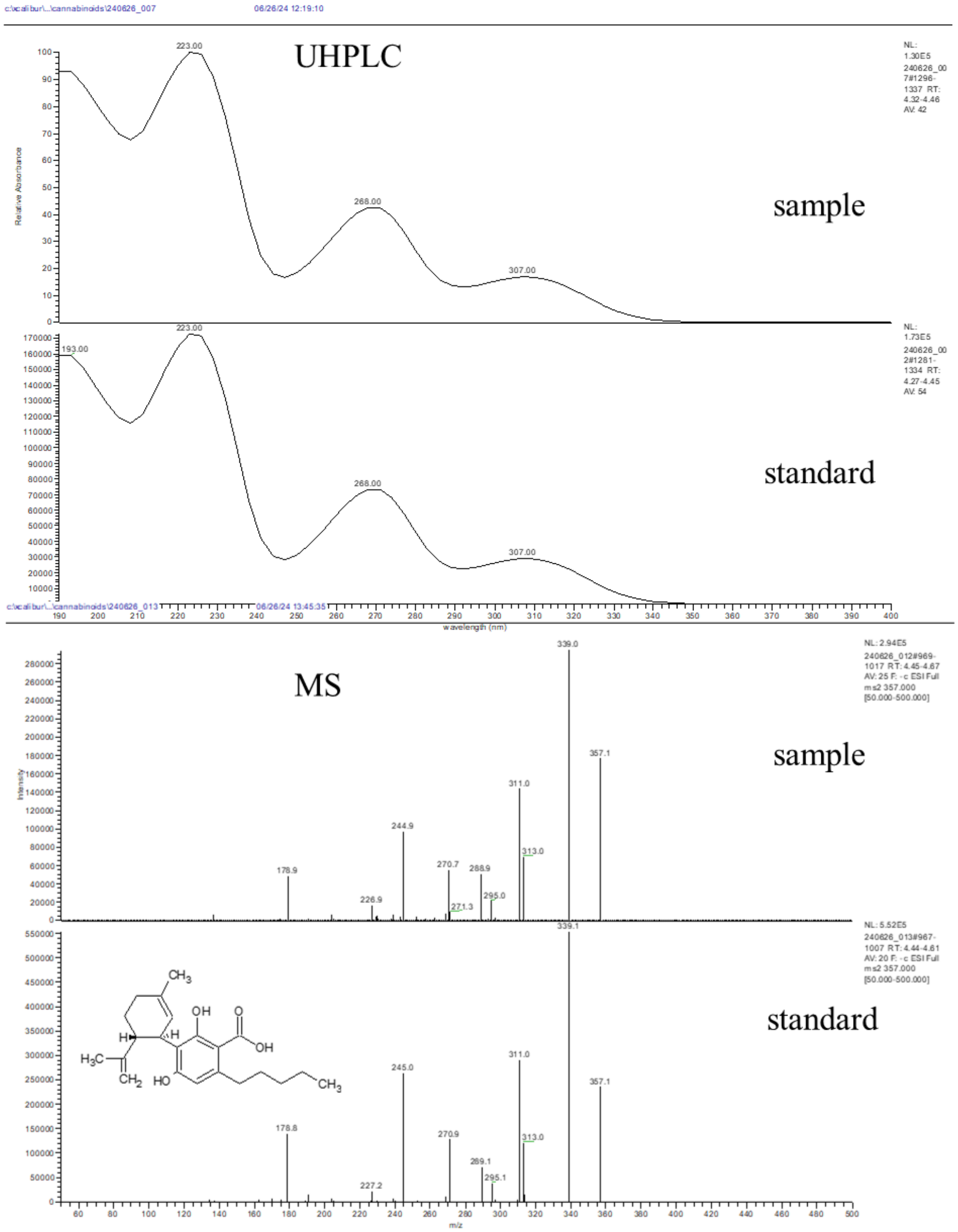

Figure S10. Identification of compound 6 (cannabidiolic acid) with UHPLC/MS.

Table S1. Column chromatography method for the separation of chloroform fraction of tetrahydrocannabinol-rich *C. sativa* extract.

Column	length 75 cm, diameter 10 cm
Stationary phase	silica gel (particle size 0.04-0.063 µm)
Mobile phase	Dichlorethane
Collected volume per fraction	100 mL
Number of fractions	178

Table S2. Column chromatography method for the separation of the chloroform fraction of cannabigerol-rich *C. sativa* extract.

Column	length 35 cm, diameter 2.5 cm
Stationary phase	silica gel (particle size 0.04-0.063 µm)
Mobile phase	Chloroform:ethyl acetate (9:1)
Collected volume per fraction	100 mL
Number of fractions	62

Table S3. Flash chromatography method for cannabidiol-rich *C. sativa* extract.

Column	length 20 cm, diameter 3.5 cm
Stationary phase	C-18 silica gel (particle size 15 µm)
Mobile phase	Methanol/water
Elution type	Gradient:
	0-10 min 100% H2O
	10-50 min gradient to 30% MeOH
	50-59 min 100% MeOH
Flow rate	25 mL/min
Collected volume per fraction	25 mL
Number of fractions	40
